# Regulation of Host Innate Immunity by Non-Coding RNAs During Dengue Virus Infection

**DOI:** 10.3389/fcimb.2020.588168

**Published:** 2020-11-30

**Authors:** Roopali Rajput, Jitender Sharma, Mahima T. Nair, Madhu Khanna, Pooja Arora, Vikas Sood

**Affiliations:** ^1^ Department of Microbiology (Virology Unit), Vallabhbhai Patel Chest Institute, University of Delhi, Delhi, India; ^2^ Department of Molecular Medicine, National Institute of Tuberculosis and Respiratory Diseases, New Delhi, India; ^3^ Department of Biochemistry, All India Institute of Medical Sciences, Bathinda, India; ^4^ Department of Zoology, Hansraj College, University of Delhi, Delhi, India; ^5^ Department of Biochemistry, School of Chemical and Life Sciences, Jamia Hamdard, New Delhi, India

**Keywords:** innate immunity, lncRNA, miRNA, sfRNA, circRNA, dengue virus, lincRNA

## Abstract

An estimated 3.9 billion individuals in 128 nations (about 40% of global population) are at risk of acquiring dengue virus infection. About 390 million cases of dengue are reported each year with higher prevalence in the developing world. A recent modeling-based report suggested that half of the population across the globe is at risk of dengue virus infection. In any given dengue outbreak, a percentage of infected population develops severe clinical manifestations, and this remains one of the “unsolved conundrums in dengue pathogenesis”. Although, host immunity and virus serotypes are known to modulate the infection, there are still certain underlying factors that play important roles in modulating dengue pathogenesis. Advanced genomics-based technologies have led to identification of regulatory roles of non-coding RNAs. Accumulating evidence strongly suggests that viruses and their hosts employ non-coding RNAs to modulate the outcome of infection in their own favor. The foremost ones seem to be the cellular microRNAs (miRNAs). Being the post-transcriptional regulators, miRNAs can be regarded as direct switches capable of turning “on” or “off” the viral replication process. Recently, role of long non-coding RNAs (lncRNAs) in modulating viral infections *via* interferon dependent or independent signaling has been recognized. Hence, we attempt to identify the “under-dog”, the non-coding RNA regulators of dengue virus infection. Such essential knowledge will enhance the understanding of dengue virus infection in holistic manner, by exposing the specific molecular targets for development of novel prophylactic, therapeutic or diagnostic strategies.

## Introduction

Dengue, a mosquito-borne viral disease, is prominent in tropical and sub-tropical regions across the world. It is caused by the dengue virus (DENV) that circulates in the form of four serotypes, DENV-1 to 4. In the past decades, the incidence of dengue has strikingly increased, such that almost half of the global population remains at risk of this infection. The annual estimates for DENV infections are between 100 and 400 million (WHO, 2020). As it is with any other communicable disease, community involvement remains the most sustainable control measure along with the effective vector control efforts. Furthermore, in conjunction to the non-pharmacological control measures, understanding of the biology of dengue is of utmost importance; in order to continue development of novel therapeutic and prophylactic strategies. While the RNAi- mediated regulation of genes has been known for quite a while now; the back-end control of gene regulation has been of much interest recently. For instance, the role of long non-coding RNAs (lncRNAs) (that themselves act as precursors to miRNAs) in modulation of important immune responses is being investigated. Also, the extent of viral non-coding RNAs in modulation of host innate immunity is also being understood. The present review is an attempt to comprehend the role of non-coding RNAs in modulation of hosts’ innate immune defense during DENV infection.

## Overview of Dengue Virus Structure and Infection

DENV has been placed in the *Flaviviridae* family under the genus *Flavivirus* and circulates worldwide (endemic in >100 countries) in the form of four serotypes, all of which are assumed to have been originated and later on independently evolved from the strains circulating in the Asian-Oceanic region (Wang et al., 2000). The virus is spherical and enveloped exhibiting the icosahedral symmetry, a lipid bilayer and a nucleocapsid core coating the positive-sense single-stranded RNA (ssRNA) genome (Kuhn et al., 2002). The viral genome (about 10,700 bp) codes for a single precursor poly-protein (approx. 3,411 amino acids long) from which the other functional viral proteins (three structural and seven non-structural) are processed. Of the three structural proteins, *viz*., capsid, precursor membrane, and envelope, the envelope glycoprotein remains the focus of interaction with the neutralizing antibodies as it is involved in receptor attachment and fusion facilitating viral entry into the host cells (Chambers et al., 1990). The precursor membrane protein along with the envelope protein forms a trimeric protrusion in immature virions’ surface (Zhang Y. et al., 2003). The capsid protein attached with the viral RNA genome is located beneath the outer protein coat and the lipid bilayer (Zhang W. et al., 2003). The non-structural proteins, *viz*., NS1, NS2a, NS2b, NS3, NS4a, NS4b, and NS5, have found to be primarily related to evasion of host’s immune responses and viral replication (Uno and Ross, 2018).

DENV can infect a variety of cell types, like, dendritic cells (DCs), endothelial cells, fibroblasts, keratinocytes, macrophage, mast cells, and monocytes (Garcia et al., 2017), and hence is known to utilize a diverse range of host surface receptors [like, heparan sulfate- lectins, DC-SIGN, mannose receptor of macrophages, lipopolysaccharide (LPS) receptor CD14, Heat-shock proteins 70 and 90, endoplasmic reticulum chaperonin GRP78, TIM-1, AXL, Claudin-1 proteins, etc.] to enter into the host cell (Cruz-Oliveira et al., 2015). Among the known target cell types, DENV antigens are expressed on cell surfaces of lymphocytes, monocytes and macrophages, as revealed by human post mortem studies. An important characteristic feature of DENV infection is that the DCs are the direct targets of profound infection by DENV, while in other viral hemorrhagic fever cases, endothelial cells are the direct targets. In studies involving human dengue cases, it has been observed that the endothelial cells of lungs and spleen express viral proteins at a relatively lower frequency, but definitely not the viral RNA (Srikiatkhachorn and Kelley, 2014). The DENV envelope glycoprotein interacts with one of such available host cell receptors to enable its entry *via* clathrin-mediated endocytosis; following which a decline in endosomal pH occurs leading to a conformational change in the virion, fusion of the membranes, and eventual release of the viral genome into the cytoplasm (Heinz and Allison, 2000).

Owing to the positive sense orientation, the viral RNA genome, once inside the host cell cytoplasm, gets readily translated into polyprotein by the host ribosome (Polacek et al., 2009). This follows cleavage of the polyprotein by both DENV and host proteases into functional viral proteins. The progeny virion assembly takes place in the golgi apparatus where an inefficient process, i.e., cleavage of precursor membrane protein by host furin protease happens (Welsch et al., 2009; Junjhon et al., 2010). The process is considered inefficient as it generates immature and partially mature virions along with the infectious mature virions that finally exit the host cell *via* exocytosis.

## Innate Immune Responses to Dengue Virus Invasion

**Figure 1:**
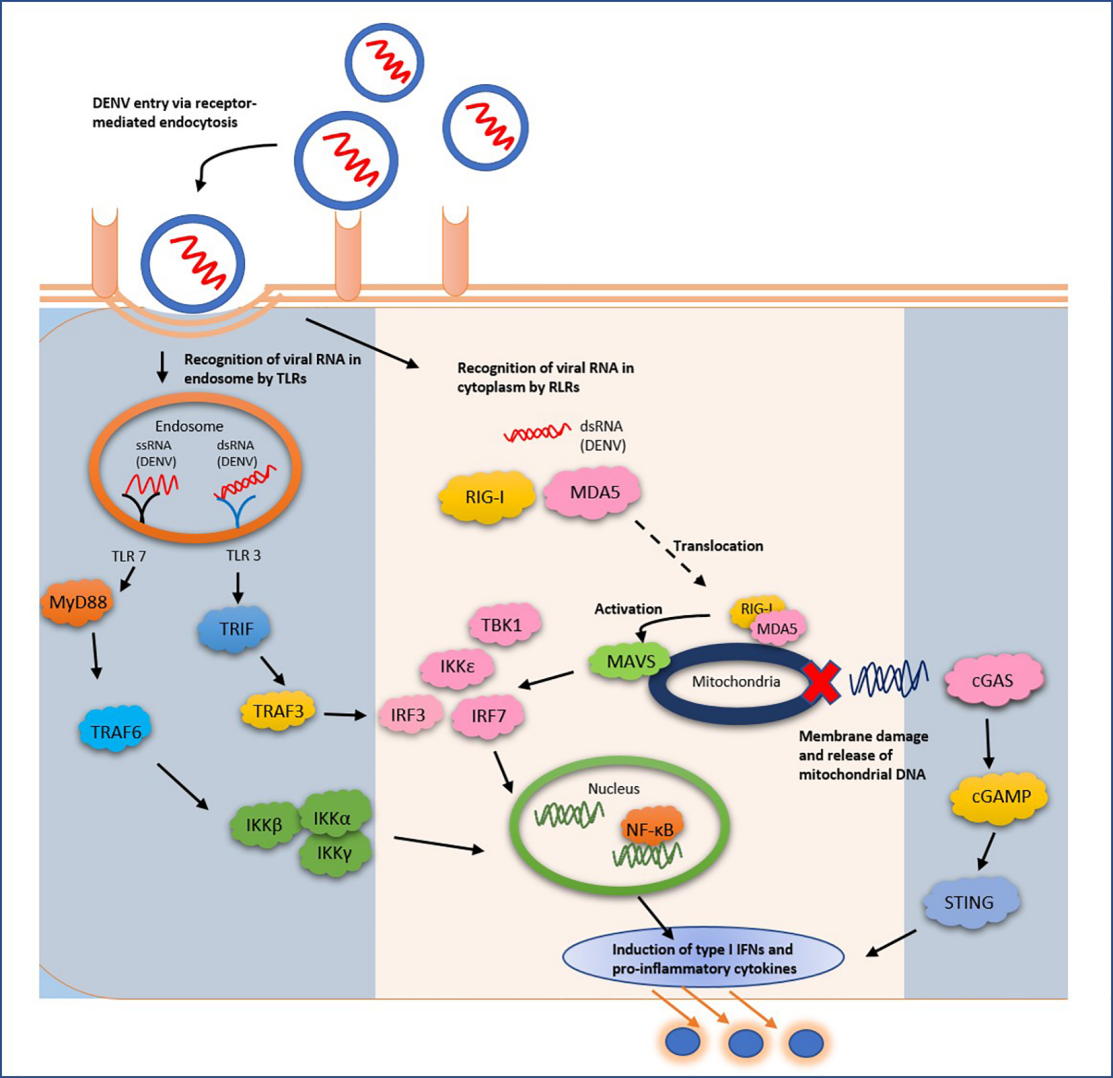
Initial establishment of anti-viral state *via* innate immune responses to DENV infection. As soon as the DENV invades host cells, the TLRs (blue panel, left) and RLRs (pink panel) recognize the viral RNA and induce a series of signals to establish a state of emergency. As described in the text, the series of signaling lead to mitochondrial damage and release of the organelle DNA (blue panel, right), furthering type I interferon responses. This antiviral state in the initial host cells is able to extend up to the adjoining cells *via* IFN signaling. Various non-coding RNAs (as described in the text) regulate different targets in the innate immune responses (demonstrated above) including the complement system pathway (not included in the above figure).

The DENV initially propagates in skin cells (keratinocytes and Langerhans cells) (Garcia et al., 2017) inducing the innate arm of the immune system. DCs, macrophages, and monocytes are quick to respond by recognition of pathogen-associated molecular patterns *via* pattern recognition receptors (PRRs) (Akira and Takeda, 2004; Loo and Gale, 2011), *viz*., cytoplasmic retinoic acid-inducible gene I (RIG-I) and melanoma differentiation-associated protein 5 (MDA5), along with endosomal Toll-like receptor 3 (TLR3) and TLR7 (**Figure 1**) (Wang et al., 2006; Nasirudeen et al., 2011). This, in turn, stimulates type I interferon responses, secretion of cytokines and chemokines, eventually establishing an antiviral state. The RIG-I and MDA5 [both of which belong to RIG-I like receptors (RLRs)], upon identification of the DENV *RNA in the cytoplasm* translocate to mitochondrial membrane, and cause stimulation of mitochondrial antiviral signaling (MAVS) protein, leading to activation of TANK-binding kinase 1 (TBK1), IκB kinase- ϵ (IKKϵ), phosphorylating IFN regulatory factors (IRF3), and IRF7. These molecules translocate into the infected cell’s nucleus and trigger production of type I IFNs (Seth et al., 2005). The TLR responses work a little differently; in a sense that the *double stranded* and *single stranded RNA* molecules of DENV are identified *in endosomes* and *DC endosomes* by TLR3 and TLR7, respectively (Baum and García-Sastre, 2010). The TLR3 functions in sync with the RLRs in establishing an antiviral state against the viral invasion (Nasirudeen et al., 2011). The activated TLR3 induces IFN-α/β-stimulating-genes (ISGs) and chemokines *via* interaction of phosphorylated TRIF (TIR-domain-containing adapter inducing IFNβ), TRAF3 (TNF-receptor-associated factor 3) and TBK1/IKKϵ (Akira and Takeda, 2004). On the other hand, TLR7 stimulates secretion of pro-inflammatory cytokines *via* the MyD88 (myeloid differentiation primary response gene 88) dependent signaling. The pathway involves TRAF6-mediated activation of nuclear factor-κB (NF-κB) (Wang et al., 2006). In another series of responses, the STING (stimulator of IFN gene) pathway gets activated by the cyclic GMP-AMP synthase (cGAS) PRR. It is important to note that the STING pathway recognizes the cytoplasmic DNA (Sun et al., 2017). During infection by the DENV, the STING pathway gets activated due to the release of mitochondrial DNA (mtDNA) into the cytoplasm, following DENV-led damage of the mitochondria (Aguirre et al., 2017), eventually causing production of type I IFNs. Under *in vitro* conditions, the presence of mtDNA has been shown to stimulate another endosomal PRR, the TLR9, which enables identification of DNA harboring non-methylated CpG motifs in human DCs (Lai et al., 2018).

Furthermore, innate immune responses have been shown to regulate severity of DENV infection. A study involving human challenge experiments revealed the role of sustained induction of IFN-gamma in acute DENV infections (Gunther et al., 2011). Similarly, the C-type lectin domain family 5, member A (CLEC5A) protein has been shown to regulate cytokine storm in dengue-infected mice model (Chen et al., 2008; Sung et al., 2019). Single cell RNA-seq of dengue-infected patients revealed that MX1 and IFIT1 were highly upregulated in DENV patients before the development of severe disease (Zanini et al., 2018).

The establishment of this initial antiviral state and the subsequent type I IFN responses somehow protect the other monocytes from the DENV threat (Diamond et al., 2000). This is attained by activation of the JAK/STAT (Janus kinase/signal transducer and activator of transcription) pathway and secretion of ISGs in adjoining cells, in order to maintain and enhance the antiviral state (Morrison and García-Sastre, 2014).

The DENV infection also attracts reaction from the complement system. As the name suggests, the mannose-binding-lectin (MBL), which is considered as an important soluble PRR of the innate immunity, binds to the mannose-harboring glycans on the DENV. This leads to activation of the MBL pathway that ultimately leads to neutralization of the DENV (Avirutnan et al., 2011) and inflammation (Fujita et al., 2004).

## Role of Non-Coding RNAs in Innate Immune Responses to DENV Infection

### Acting as Antivirals

Apart from the traditional innate immunity-led defense against the DENV infection, host or viral non-coding RNAs are also shown to project protection. During viral infection, the host miRNA along with the RNA-induced silencing complex (RISC) identifies and degrades the viral RNA (Jeang, 2012). An *in vitro* study demonstrated that the DENV infection leads to suppression of the host RNAi agents, *viz*., Dicer, Drosha, Ago1, Ago2; and that an inhibition of these RNAi factors led to elevated DENV titer in the human hepatocellular carcinoma “Huh7” cells. The researchers found that the transmembrane domain 3 (TMD3) of the non-structural 4b protein caused this suppression of RNAi during infection by any of the four DENV serotypes (Kakumani et al., 2013). Similarly, the role of hsa-mir-126-5p in negative regulation of DENV infection was also studied (Kakumani et al., 2016). In a study, it was shown that transfection of miR-126-5p (an miRNA mimic of has-126-5p) in Huh-7 cells led to increase in hsa-126-5p levels by 3.41 fold and a simultaneous 70% decline in vRNA levels after 24 h of virus infection (Kakumani et al., 2016). Different miRNAs, *viz*., let-c, miRNA-30e*, and miRNA-126-5p, are reportedly modulated during DENV infection. MicroRNAs also identify or be identified by the TLRs or the RLRs (RIG-I and MDA5), eventually modulating the innate immunity to DENV infection (Urcuqui-Inchima et al., 2017). In a microarray expression analysis, 89 dysregulated miRNAs were found to be associated with 499 potential targets during treatment of critical DENV infection. Among the top-hit targets, DDX3X (DEAD-Box Helicase 3, X-Linked) and PTEN (Phosphatase and Tensin Homolog) were speculated to have important roles in DENV infection. While, the DDX3X is an important regulator of cell proliferation and is able to induce IFN promoter branches during DENV-infected cells, indicating its antiviral effect being modulated by respective miRNA (Shahen et al., 2018), PTEN is known to exhibit antiviral effect against dengue (Wagstaff et al., 2012). Likewise, miR-30e* is also known to exert antiviral impact by furthering the production of IFN-β *via* the NF-κB pathway during dengue (Zhu et al., 2014). A small RNA-seq analysis revealed differential expression of five miRNAs in DENV-infected and -exposed but non-infected human primary macrophages. The DENV-non-infected macrophages expressed elevated levels of miR-3614-5p, which acted as an antiviral agent by targeting a DENV pro-viral protein, adenosine deaminase acting on RNA 1 (ADAR1) (Diosa-Toro et al., 2017).

In another interesting analysis, it was found that over-expression of hsa-miR-133a negatively regulates replication of all four serotypes of DENV (Castillo et al., 2016) in Vero cells, probably *via* the polypyrimidine tract binding protein (PTB). The PTB is known to have role in IRES-independent translation of viral/cellular RNA. In case of dengue, PTB binds to the 3′-UTR of the viral genome and furthers the viral RNA replication, probably by acting as a RNA helicase (De Nova-Ocampo et al., 2002). The miR-133a was speculated to target PTB specifically during the initial hours of DENV infection in the Vero cells. As a counter mechanism, all four serotypes of DENV suppress the endogenous levels of miR-133a so as to allow high expression of PTB leading to viral replication during about 12 h of infection (Castillo et al., 2016), pointing towards negative regulation of DENV by miR-133a. Such an interplay highlights important role of non-coding RNA mediated modulation of dengue.

Overexpression of some of the other non-coding RNAs, *viz*., miR-548g-3p (Wen et al., 2015), miR-484 and miR-744 (Castrillón-Betancur and Urcuqui-Inchima, 2017), inhibit replication of all the four DENV serotypes. MiR-548g-3p interferes with the expression of viral proteins as well. The miR-548g-3p targets the 5′-UTR of DENV genome, specifically at the stem loop A promoter region and leads to suppression of virus propagation in an interferon-independent manner (Wen et al., 2015).

### Acting as Pro-Virals

The best known pro-viral miRNA, which is highly expressed in monocytes during dengue, is the miR-146a (Wu et al., 2013). MiR-146a is a known regulator of innate immunity, inflammatory responses and viral replication (Li et al., 2010). Wu and colleagues hypothesized that the miR-146a elevates replication of DENV serotype-2 by decreasing the host IFN-β production *via* targeting tumor necrosis factor receptor (TNFR)–associated factor 6 (TRAF6) (Wu et al., 2013). However, an overexpression of the miR-146a substantially inhibited DENV-2 *via* autophagy (Pu et al., 2017). It was also found that an antagonist, LNA-antagomir-146a was able to suppress the miR-146a effect and restore the host IFN activity (important host antiviral defence mechanism) against the virus. In a later study, the serum levels of miR-146a seemed to be reduced along with a negative correlation with serum AST/ALT levels in dengue subjects. This indicated a possible role of miR-146a in liver inflammation (Ouyang et al., 2016). Similarly, miR-21 is also a known inhibitor of pro-inflammatory response (Sheedy, 2015) and hence its expression is upregulated during dengue Ouyang et al., 2016). The miR-21 also augments DENV-2 replication in HepG2 cells (Kanokudom et al., 2017). The miR-21 is thought to target NS1 protein of DENV-2 (Wong et al., 2020), which is known to evade the complement innate immune responses by blocking the classical pathway C3 convertase (Avirutnan et al., 2010), and also escape the MBL-mediated neutralization (Thiemmeca et al., 2016).

Apart from the host factors, viral non-structural 3 (NS3) protein is also known to regulate biogenesis and function of host miRNAs in human embryonic kidney (HEK) 293T cells. Amazingly, the negative regulation of host miRNAs exerted by the DENV NS3 was found to be stage-specific to enable up-regulation of the viral host factors, *viz*., up-regulation of TAZ (tafazzin) and SYNGR1 (synaptogyrin 1), facilitating DENV replication (Kakumani et al., 2015).

A high throughput RNA sequencing analysis revealed a significant up- or down-regulation of various lncRNAs in L-02 liver cells post DENV infection. Upon analysis of the lncRNA-mRNA co-expression networks, 68 and 50 interacting nodes were identified by infection of DENV serotype 1 and 2, respectively. The differentially expressed lncRNAs were observed to be potential precursors to mature miRNAs, *viz*., hsa-mir-29b-2, -29c, -22, -1268a, and -3648, and were found to be associated with various biological processes in host cells during DENV infection, such as, biosynthesis, nucleic acid related processes, estrogen signaling, cytoskeleton reorganization, stimulation of apoptosis, to point a few (Wang et al., 2017). In another genome-wide profiling analysis of mRNA and lncRNA expression during dengue and dengue haemorrhagic fever (DHF), 215 and 225 lncRNAs were differentially expressed in dengue and DHF, respectively. Upon, functional analysis, *MAGED1*,* STAT1*, and *IL12A* genes were found to be significantly dysregulated. *MAGED1* has been linked to severe dengue (Silva et al., 2013), *STAT1* is known to supplement protective antiviral interferon responses in presence of schisandrin A against DENV replication (Yu et al., 2017), and *IL2A* stimulates IFN‐γ production and differentiation of Th1 and Th2 cells (Lamont and Adorini, 1996). The role of lncRNA in dengue disease progression has also been studied. RNA sequencing was used to investigate and compare the expression profiles of various lncRNAs and protein-coding genes in samples collected from dengue patients exhibiting different extent of severity and in samples from patients presenting with other febrile illnesses. Nuclear Enriched Abundant Transcript 1 (NEAT1), which is a non-coding RNA, and the coding gene Interferon alpha-inducible protein 27 (IFI27) were highly co-expressed and negatively associated with the degree of dengue severity (Pandey et al., 2017). NEAT1 is an important regulator of innate immunity, as it affects the transcriptional regulation of several anti-viral genes (Ahmed and Liu, 2018). This might explain NEAT1 as a differentiating bio-marker of severe dengue from dengue infection (Pandey et al., 2017). In another study, the transcript levels of long intergenic non-coding RNA (lincRNA) in DENV2 infected mosquitos showed 32% decrease in lincRNA post infection in *Aedes aegypti* whereas majority of lincRNAs were over-expressed. The transcription levels of 72 lincRNAs were up-regulated post infection. Supporting the role of certain lincRNA through RNAi mediated silencing of lincRNA_1317 in Aa20 cells and then infected by DENV2 increased the viral replication and infection progression proving that this lincRNA is important for anti-viral response, it was also over expressed in infected mosquitos rather than non-infected ones (Etebari et al., 2016).

Another set of interesting non-coding RNAs are the circular RNAs (circRNAs) that lack free 5′ and 3′ ends and have a closed loop instead. CircRNAs are referred to as transcriptional products that are developmentally regulated at tissue or cell type levels (Barrett and Salzman, 2016). The exact role of circRNAs is not clear at present (Barrett and Salzman, 2016). With respect to human diseases, it has been shown that the levels of certain circRNA vary as per the disease profile. For instance, expression of hsa_circ_0015962 and miR-133b were reportedly elevated in post-treatment group than the pre-treatment group of dengue fever patients. The treatment, here, refers to administration of pain relievers, intra-venous fluids and critical care at hospital to severe dengue patients. Expression of hsa_circ_0006459 and miR-4683 was found to be lower in the post-treatment group than in the pre-treatment group. Furthermore, it was demonstrated that the hsa_circ_0015962 binds and negatively modulates the expression of miR-4683, while the hsa_circ_0006459 targets and negatively regulates miR-133b (He et al., 2019).

In addition to the non-coding RNAs expressed by host, some are expressed by viruses also. During replication of flaviviruses, the uncapped genomes are digested by the host 5′ to 3′ exoribonuclease, however, the process gets stopped when a pseudoknot RNA structure is encountered in the 3′ UTR region. This results in formation of about 0.3 to 0.5 kb sized sub-genomic flavivirus RNA (sfRNA). Elevated levels of sfRNA lead to inhibition of TRIM25 gene and can deactivate RNA binding proteins which are crucial for innate immunity. Reduced guide RNA (gRNA) levels may cause lower stimulation of RIG-I/MDA5, which are the initial drivers of innate IFN responses. Briefly, the DENV-2 clade (PR-2B) sfRNA interacts with TRIM25 (which is an E3 ubiquitin ligase and also an RNA-binding protein), averting ubiquitin-specific peptidase 15 (USP15) to deubiquitinylate TRIM25. This, in turn, stops TRIM25 to polyubiquitinate RIG-I causing a drop in IFN production. This can ultimately render several host cells susceptible to DENV causing viremia (Manokaran et al., 2015). The available literature indicates that the higher sfRNA production and also the structure or sequence of the sfRNA may have important implications on epidemiological fitness of DENV-2. For instance, the 1994 dengue outbreak in Puerto Rico can be understood in terms of the sfRNA-gRNA ratios. Higher levels of the sfRNA and the decreased levels of gRNA lead to inhibition of TRIM25 and suppressed trigger of RIG-I/MDA5 responses, respectively. And it is quite well-known that the early interferon responses are important to combat establishment of the viral infection and halt the mosquito-borne transmission.

Further, DENV also encodes functional viral small RNAs (vsRNAs). One such vsRNA, DENV-vsRNA-5, was found to act similar to miRNA and exhibited important role in “autoregulation” of virus replication. DENV-vsRNA-5 targets the virus nonstructural protein 1 (NS1) gene and suppresses DENV-1, -2, and -4 replication in mosquito cells (Hussain and Asgari, 2014). This is an interesting finding of virus autoregulation mechanisms and may be of interest for further exploration and use of small RNAs as antiviral agents.

## Differentiation of Mild vs. Severe Dengue

Severe dengue is reported to be strongly linked with “cytokine storm”, a condition when the pro- and anti-inflammatory mediators get imbalanced. SOCS family of proteins have been known to negatively regulate various signaling pathways. SOCS1, a controller of several cytokines, is negatively regulated by miR-150 during Dengue Haemorrhagic Fever (DHF) (Chen et al., 2014). Chen and colleagues observed that whereas miR-150 was highly induced in DHF patients, levels of SOCS1 were reduced in the same thereby pointing toward the reciprocal interplay among SOCS1 and miR-150. Another study further confirmed these findings and observed enhanced levels of miR-150 in severe dengue patients (Hapugaswatta et al., 2020). SOCS1 was further identified in patients with acute dengue infections (Hoang et al., 2010). The reports suggest that SOCS-1 protein is dysregulated in dengue patients and might be one of the contributing factors toward cytokine storm during dengue pathogenesis. Decreased expression of miR-106b, miR-20a, and miR-30b during DENV-2 is also thought to elevate production of pro-inflammatory cytokines (Qi et al., 2013). MiR-let-7e possibly regulates IL-6 and CCL3, while miR-451 and miR-4279 are deemed as modulators of CCL5 and CXCL1 expression. Low miR-106b expression might lead to increased secretion of CCL5, which is one of the important host factors during viral replication, especially DENV2 infection. Also, the chemokine CCL5 is rapidly produced by mast cells when activated by the DENV-antibody complexes (Brown et al., 2012) and also related to DENV- triggered hepatic dysfunction (Conceicao et al., 2010).

**Figure 2:**
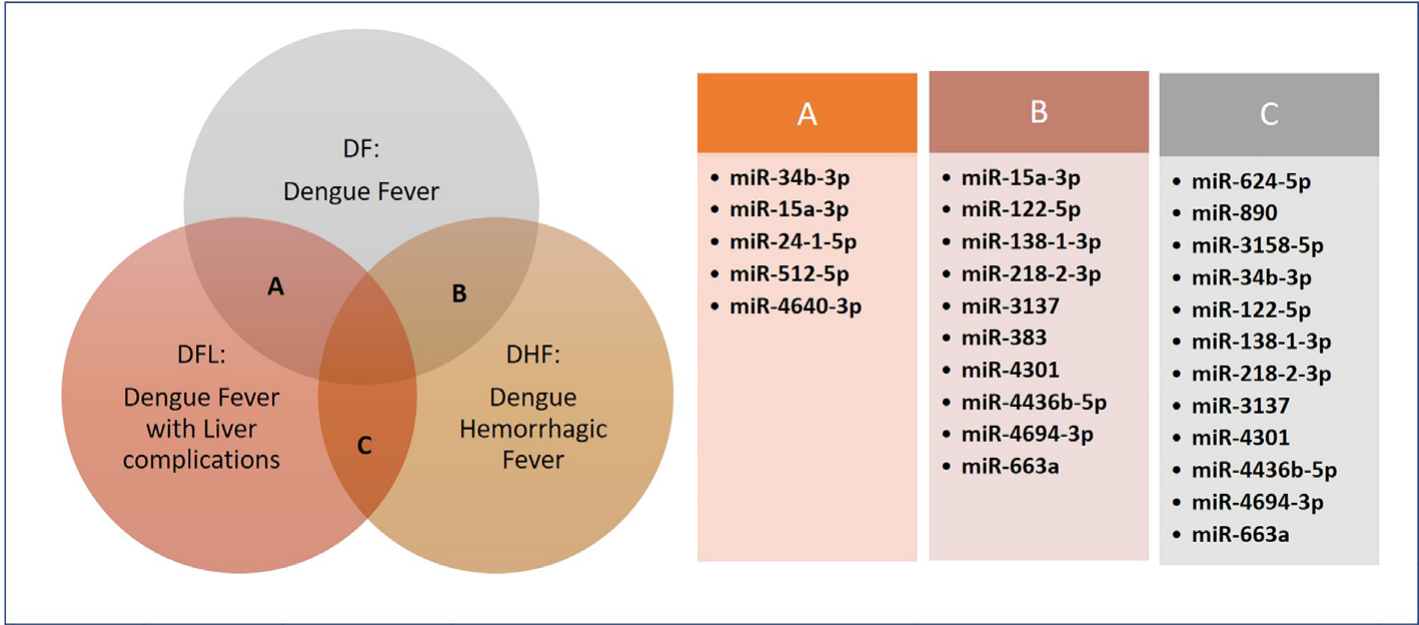
Differentially expressed miRNAs in mild vs. severe dengue conditions.

Some interesting studies have been aimed at deciphering the role of non-coding RNAs in distinguishing mild dengue with severe dengue and the related complications. One such interesting study involved comparison of miRNA profiling in blood specimens of dengue and influenza patients. The two categories of diseased cases were taken to enable identification of unique miRNA signatures of dengue. As speculated, of the 106 dysregulated miRNAs associated with acute dengue, 14 miRNAs displayed similar expression profiles in both the diseases, while 12 were unique to acute dengue, i.e., within 0-4 days of the illness. Upon functional analysis, these 12 miRNAs (hsa-miR-450b-5p, -491-5p, -499a-3p, -512-5p, -615-5p, -624-5p, -892b, -1204, -1225-5p, -3121-3p, -4259, and -4327) were found to regulate the P13K/AKT survival pathway. Among these, hsa-miR-1204, 491-5p, and 512-5p seemed to have important roles in apoptosis, P13K/AKT pathway and indirect modulation [via NOD2 (nucleotide-binding oligomerization domain containing 2) gene] of NF-kB, JNK, and MAPK pathways, interleukins, and cytokines, respectively. Moreover, 17 miRNAs were also identified that specified complications arising from dengue, for instance, liver complications, abdominal pain, and capillary leak excluding shock (**Figure 2**) (Tambyah et al., 2016). The miRNAs hsa-miR-24-1-5p, miR-512-5p, and miR-4640-3p were significantly varied in expression profiles between dengue fever (DF) and dengue with liver complications (DFL) patients. Also, the hsa-miR-383 showed 6.3 fold upregulated levels in DF than in the dengue fever with clinical fluid accumulation (DHF) subjects. The expression of miRNAs, *viz*., hsa-miR-624-5p, miR-890 and miR-3158-5p, varied among the severe dengue DFL and DHF categories also. An interesting finding was the expression of hsa-miR-15a-3p, which was highly (48 fold; p < 0.0005) downregulated in DF subjects, but only mildly (approx. 5-fold) downregulated severe dengue (DFL and DHF) patients. These observations are crucial indicators that the miRNAs and their extent of dysregulation can be important differentiators of mild and severe dengue (Tambyah et al., 2016).

The previously discussed antiviral miR-744 (Castrillón-Betancur and Urcuqui-Inchima, 2017) may also be a differentiating marker of mild vs. severe dengue, as it targets an important inflammation regulatory protein, TGF-β1 (Martin et al., 2011). TGF-β1 gene polymorphism (−509 CC genotype) is known genetic marker for DHF susceptibility and high viral load (Chen et al., 2009).

A recently published report utilized NGS based approaches to identify circulating miRNAs among DENV infected patients. The study could successfully correlate the dysregulation of several miRNAs among mild and severe dengue patients. (Saini et al., 2020). Specifically, the expression profile of hsa-miR-122-5p in plasma specimens was found to be an important differentiator of dengue infection stage. The miRNA could also demarcate between dengue-negative subjects from other febrile illnesses.

## Conclusion

The endemic state of dengue in many tropical and sub-tropical nations imposes a serious risk for the other similar climate areas. The symptoms, generally, start surfacing after 4–5 days of incubation period. Since its origin, still there is no effective treatment or vaccine for dengue. Hence, modulation of the molecular regulators, like the various dysregulated non-coding RNAs seem to be lucrative therapeutic option. Further investigations in this area would definitely garner confidence and effective practical use of this approach.

## Author Contributions

RR and VS conceived and designed the study. RR collected the data, prepared the initial draft and figure, proofread, revised, and approved the final manuscript. JS provided substantial intellectual input, proofread and revised the manuscript, and edited the figure for final approval of the manuscript. MN collected data for initial draft and provided intellectual inputs. MK proofread and provided critical inputs. PA proofread and contributed to the manuscript’s structure. VS conceived the work, proofread, provided substantial intellectual inputs, and approved the final manuscript. All authors contributed to the article and approved the submitted version.

## Funding

VS received research grant from the University Grants Commission (UGC), Govt. of India & Promotion of University Research and Scientific Excellence (PURSE), Department of Science & Technology (DST), Govt. of India. JS received institutional support from AIIMS, Bathinda, Punjab, India.

## Conflict of Interest

The authors declare that the research was conducted in the absence of any commercial or financial relationships that could be construed as a potential conflict of interest.
